# Patterns of viral pathogens causing upper respiratory tract infections among symptomatic children in Mwanza, Tanzania

**DOI:** 10.1038/s41598-020-74555-2

**Published:** 2020-10-28

**Authors:** Elizabeth Kwiyolecha, Britta Groendahl, Bernard Okamo, Neema Kayange, Festo Manyama, Benson R. Kidenya, Dina C. Mahamba, Delfina R. Msanga, Stephan Gehring, Mtebe Majigo, Stephen E. Mshana, Mariam M. Mirambo

**Affiliations:** 1 Department of Pediatrics & Child Health, Catholic University of Health and Allied Sciences, P.O. Box 1464, Mwanza, Tanzania; 2 Department of Pediatrics, University Medical Center of the Johannes Gutenberg University Mainz, Mainz, Germany; 3 Department of Biochemistry and Molecular Biology, Weill Bugando School of Medicine, Catholic University of Health and Allied Sciences, P.O. Box 1464, Mwanza, Tanzania; 4 Department of Microbiology and Immunology, Muhimbili University of Health and Allied Sciences, P.O. Box 65001, Dar es Salaam, Tanzania; 5 Department of Microbiology and Immunology, Weill Bugando School of Medicine, Catholic University of Health and Allied Sciences, P.O. Box 1464, Mwanza, Tanzania

**Keywords:** Infectious diseases, Respiratory tract diseases

## Abstract

Upper-respiratory tract infections (URTI) are the leading causes of childhood morbidities. This study investigated etiologies and patterns of URTI among children in Mwanza, Tanzania. A cross-sectional study involving 339 children was conducted between October-2017 and February-2018. Children with features suggestive of URTI such as nasal congestion, dry cough, painful swallowing and nasal discharge with/without fever were enrolled. Pathogens were detected from nasopharyngeal and ear-swabs by multiplex-PCR and culture respectively. Full blood count and C-reactive protein analysis were also done. The median age was 16 (IQR: 8–34) months. Majority (82.3%) had fever and nasal-congestion (65.5%). Rhinitis (55.9%) was the commonest diagnosis followed by pharyngitis (19.5%). Viruses were isolated in 46% of children, the commonest being Rhinoviruses (23.9%). Nineteen percent of children had more than 2 viruses; Rhinovirus and Enterovirus being the commonest combination. The commonest bacteria isolated from ears were *Staphylococcus aureus* and *Pseudomonas aeruginosa.* Children with viral pathogens had significantly right shift of lymphocytes (73%—sensitivity). Majority (257/339) of children were symptoms free on eighth day. Viruses are the commonest cause of URTI with Rhinitis being the common diagnosis. Rapid diagnostic assays for URTI pathogens are urgently needed in low-income countries to reduce unnecessary antibiotic prescriptions which is associated with antibiotic resistance.

## Introduction

Respiratory tract infections (RTIs) account for an estimated 3.9 million deaths annually among children worldwide with 42% of these deaths occurring in Africa^1–3^. In high income countries, up to 25% of children under 1 year of age and up to 18% of children aged 1 to 4 years develop RTIs^4,5^. These illnesses range from mild to severe and life-threatening illness accounting for over two million childhood deaths annually worldwide^2,6,7^. Respiratory tract infections involving just the upper respiratory tract usually are self-limited disease requiring only supportive management. Despite it being a less severe illness, it has emerged to be a major cause of childhood morbidity with a high cost to the society, and occasionally associated with serious sequelae. In high income countries, these infections are caused by several families of virus including the newly discovered Bocavirus^1,2^. Common viral agents which have been linked to RTIs include Rhinoviruses which accounts for 30%, Respiratory syncytial virus, influenza virus, Parainfluenza viruses, Human metapneumovirus and Adenoviruses accounting for 35% of RTI with about 10% being due to Coronaviruses^8^. Bacterial upper respiratory infections are mainly due to *Streptococcus pneumoniae*^9^, *Haemophilus influenzae* and *Moraxella catarrhalis* accounting to 90% of bacterial causes^10^.

Epidemiology of URTIs, particularly in Africa, remains poorly understood and consequently underappreciated. Despite being the commonest cause of preschool absenteeism, frequent visits to health care facilities and major reason for irrational antibiotics prescriptions there is paucity of data on the magnitude of these infections in many low- and middle-income countries. In Tanzania, there is only one study that has documented the viral etiologies of RTI^3^. Therefore, there is a paramount need to establish information on the common etiologies of RTIs in Tanzania, the information that can stimulate further studies and possible control interventions including introduction of cheap and reliable methods to detect these pathogens in clinical settings.

In addition due to increased use of antibiotic without a support of a diagnostic test in the treatment of URTI as observed in number of previous studies^11–13^, make the availability of epidemiological data on the patterns of etiology of URTI of paramount important. Overuse of antibiotics without prescriptions for URTI is widespread in developing countries^14^, this is partially contributed by lacking of data on the etiologies of URTIs. Therefore, these data are relevant to clinicians in developing countries and policy makers in order to invest on the improved diagnostic facilities and reduce antibiotic prescriptions for URTIs.

## Methods

### Study area, design and study population

A cross sectional hospital based study involving 339 children aged 1–59 months presenting with RTI symptoms was conducted from October 2017 to February 2018 in the city of Mwanza, Tanzania. The study was conducted in two health facilities namely: Buzuruga Health Center (BHC), and Nyamagana District Hospital (NDH). These are public health facilities providing free services to children below 5 years of age.

### Selection criteria

The study included all children presented with nasal congestion or runny nose, hoarseness of voice with dry cough, painful swallowing with tender cervical lymph nodes and enlarged tonsils on examination, ear pain or ear discharge, nasal discharge with or without fever (axilla body temp of 37.5 °C and above).

In this study we defined: *Pharyngitis* as painful swallowing dry cough, plus or minus hoarseness of the voice (sore throat), *Tonsillitis* as painful swallowing with tender cervical lymph nodes and enlarged tonsils(primarily tonsillar inflammation) and *Rhinitis* as presence of one or more symptoms including sneezing, itching, nasal congestion, and rhinorrhea.

### Sample size estimation and sampling procedures

Sample size was calculated using Yamane Taro (1967) with precision level of 5%. The minimum sample size estimated was 270 children. However, a total of 339 children were enrolled. All children who met the inclusion criteria were serially enrolled until the desired sample size was attained.

### Data collection and sample collection

Sociodemographic and clinical information were collected using pretested structured data collection tool. Nasopharyngeal swabs (COPAN DIAGNOSTICS INC. USA, Canada) were obtained as previously described^15^ by inserting the swab into one nostril straight back along the floor of the nasal passage until reaching the nasopharynx. The swabs were rotated gently for 5–10 s to loosen the epithelial cells and collect the sample. The swabs were then inserted into viral transport medium and stored at − 80 °C until processing. In children presenting with ear discharge; ear swabs were collected using flexible shaft swab via an auditory speculum in case of inner ear while a sample from outer ear was obtained by firmly rotating swab in outer canal. Swabs were immediately taken to the laboratory for bacterial culture and sensitivity. For each consenting participant, about 4 mls of blood was also collected for blood cell counts and quantitative C reactive protein analysis. A thorough general and physical examination was performed to all enrolled children to establish clinical features.

All children were managed as per local hospitals protocol. Patients were followed two times; the first follow up was on the 4th day, where these patients were fully examined and their full blood count, CRP and ear swab culture results were revealed. In case of positive ear swab culture treatment was changed based on susceptibility patterns. The second follow up was done on the 8th day as clinical review to evaluate for disease progression.

### Laboratory procedures

Ear swab specimens were inoculated onto Chocolate agar, blood agar (BA) and MacConkey agar (MCA) plates and incubated aerobically at 37 °C for 24–48 h. In-house biochemical identification tests were used to identify isolated bacteria to their species level^16^. The identified isolates were tested for antimicrobial susceptibility following CLSI guidelines, The tested antibiotic discs included: amikacin (30 µg), gentamicin (10 µg), erythromycin (15 µg), vancomycin (30 µg), clindamycin (2 µg), ciprofloxacin (5 µg) which were used for gram positive bacteria and ampicillin (10 µg), ceftazidime (30 µg), meropenem (10 µg), amikacin (30 µg), piperacillin-tazobactam (100/10 µg) and ciprofloxacin (5 µg) for gram negative bacteria^17^. Bacterial isolates obtained were inoculated into Brain Heart Infusion broth with 20% Glycerine and stored at – 80 °C freezer. *E. coli* ATCC 25992 and *Staphylococcus aureus* ATCC 25923 were used as control strains.

Nasopharyngeal swabs were transported to Mainz University Germany and were tested to detect Enterovirus (EV), Influenza virus type A (IVA), Influenza virus type B (IVB), Respiratory syncytial virus (RSV), Parainfluenza virus type 1 (PIV1), Parainfluenza virus type2 (PIV2), Parainfluenza virus type 3 (PIV3), Parainfluenza virus type 4 (PIV4), Adenovirus (AV), Rhinovirus (RV), Human metapneumovirus (MPV), Coronavirus (CV), Bocavirus, *Mycoplasma pneumoniae* (Mpn), *Chlamydophila pneumoniae* (Cpn), *Bordetella pertussis* (Bp), *Bordetella parapertussis* (Bpp) and *Legionella pneumophila* (Lpn) using multiplex PCR as previously described^18^.

Blood samples were quantitatively tested for C-reactive protein following manufacturer instructions (Medical Instruments Co., Ltd, Shanghai, China). Blood in EDTA container (BD Vacutainer, Nairobi, Kenya) was used to estimate complete blood count (FBC) using hematological analyzer (Beckman coulter (UK) LTD)^19^.

### Statistical data analysis

Data entry was done using Microsoft excel then exported to STATA version 13 for analysis. Continuous variables (age and temperature) were summarized using median with interquartile ranges. Categorical variables (sex and level of education) were summarized using frequency and proportions. To determine the utility of FBC, and CRP in the determination of causative agents among children below 5 years of age, a 2 by 2 table and receiver operating curve (ROC) characteristic analysis were used to determine the sensitivity, specificity, positive and negative predictive values. Children presenting with symptoms for more than 7 days were classified as having chronic illness. A child who had no any symptom on day eight of follow was declared cure.

### Ethical approval and consent to participate

The approval for conducting the research was sought from the Joint CUHAS/BMC research ethics and review with ethical clearance number: CREC/255/2017. Permission to conduct the study was also sought from the Pediatrics departments at BHC and NDH. The aim and importance of the study was explained to parents/caretakers before enrollment of children to the study, followed by a signed informed consent by the parent/caretaker. All information regarding the patient remained confidential. Patient’s records were kept such that the identity of the patient was not disclosed. For those who refused to participate, were provided with services similar to the participants and had equal chance to treatment regardless of their inclusion status. All methods were carried out in accordance with relevant guidelines and regulations.

## Results

### Socio-demographic characteristics of study participants

The median age of the enrolled children was 16 (IQR: 8–34) months. The slightly majority 200 (59%) of children were seen at BHC and the slightly majority 198 (58.4) were below two years of age (Table 1). All except one child had received at least one dose of pneumococcal and Hib vaccine.

**Table 1:** Socio-demographic distribution for under five children with URTI in Mwanza city.

Characteristics	Number (n)	Percent (%)
Gender		
Male	200	59
Female	139	41
Age (months)		
1–23	198	58.4
24–59	141	41.6
Caregiver’s education level		
None	28	8.3
Primary	222	65.5
Secondary and above	89	26.2
Caregiver occupation		
Employed	22	6.5
Traders	255	75.2
Unemployed	62	18.3
Family size*		
Small	209	61.7
Large	129	38.3
Duration of illness prior to presentation		
1–7 days	295	87.0
> 7 days	44	13.0
Exposure to smoke (indoor cooking using charcoal/wood)		
Yes	314	92.6
No	25	7.4
Animal keeping		
Yes	14	4.8
No	325	95.2
History of antibiotic use during this illness		
Yes	156	46.0
No	183	54.0

### Clinical findings and patterns of URTI among enrolled children with URTI symptoms

**Figure 1:**
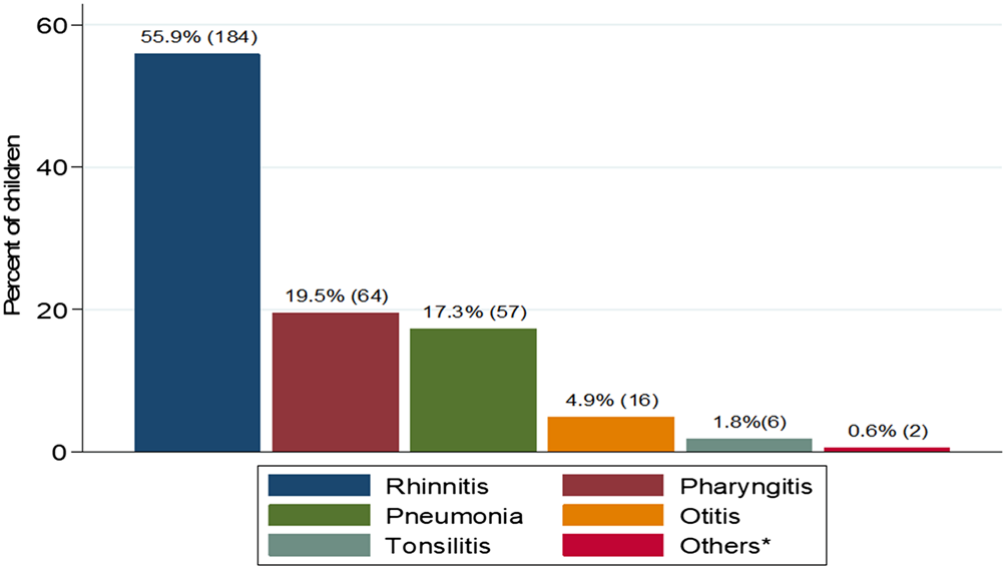
Patterns of UTRI among under five children in Mwanza city.

Among the enrolled children with URTI, the majority presented with fever 279 (82.3%) and 222 (65.5%) presented with nasal discharge. Few children 47 (13.9%) presented with abnormal chest findings (features suggestive of pneumonia) on physical examination. About 295 (87%) of the total population presented with history of illness ranging from 1 to 7 days and were classified as acute illness in this study (Table 2). The slightly majority 184 (55.9%) of children presented with rhinitis (Fig. 1).

**Table 2:** Clinical presentations of upper respiratory tract infections.

Clinical presentation	Number (n)	Percent (%)
Fever		
Yes	279	82.3
No	60	17.7
Cough		
Dry	150	44.3
Wet	39	11.5
Nasal discharge		
Yes	222	65.5
No	117	34.5
Nasal congestion		
Yes	214	63.1
No	125	36.9
Ear pain		
Yes	12	3.5
No	327	96.5
Ear discharge		
Yes	14	4.1
No	325	95.8
Rapid breathing		
Yes	36	89.4
No	303	10.6
Chest findings		
Normal	292	86.1
Abnormal	47	13.9
Tonsils examination		
Normal size	286	84.4
Enlarged	53	15.6

### Etiologies of upper respiratory tract Infections among children below 5 years of age

**Figure 2:**
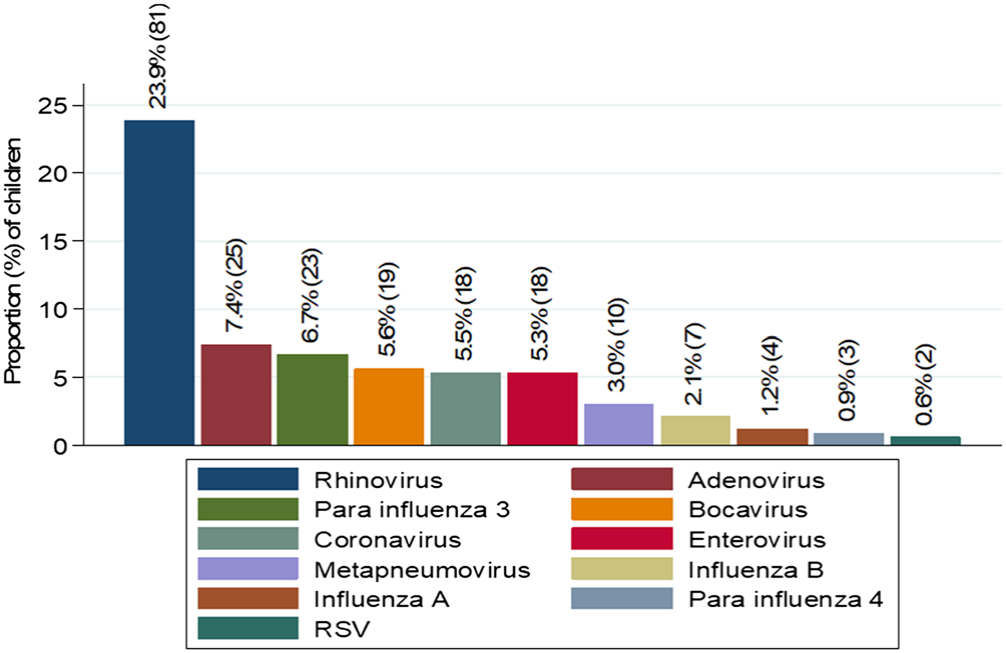
Viral pathogens isolated in the children under 5 years with URTI in Mwanza city.

Among 339 enrolled children, 159 (46.9%) children had viral pathogens detected. The commonly identified viruses were Rhinoviruses and Adenoviruses (Fig. 2). However, 40/339 (11.8%) had mixed viral infections. Only 3/339 (0.9%) children had both bacterial and viral infections. Out of 14 patients who underwent ear investigation, 11 (78.5%) yielded pathogenic bacteria. The commonest bacteria detected were *S. aureus* which was detected in 5 patients, *P. aeruginosa* in 2 patients. Other bacteria detected one each were *S. pneumoniae*, *Providence* spp. *K. pneumoniae* and *S. marcescens*. Among 159 children with viral pathogens, 136 (85.5%) presented with acute illness while 14.5% had chronic illness.

### Proportion of children with disappearance of symptoms on day eight of the illness

Out of 339, 289 were followed to determine disappearance of URTIs symptoms and 50 were loss to follow up. Majority of them 214/289 (74.0%), presented with less symptoms on the fourth day compared to the first day when they were seen at the hospital. During their second follow up visit the majority of them 257/289 (88.9%) were free from the initial symptoms and were declared cured while 32/289 (11.1%) had mild symptoms.

### Utility of lymphocytes, neutrophils and CRP in determination of etiologies of URTI among under five children in Mwanza city

**Figure 3:**
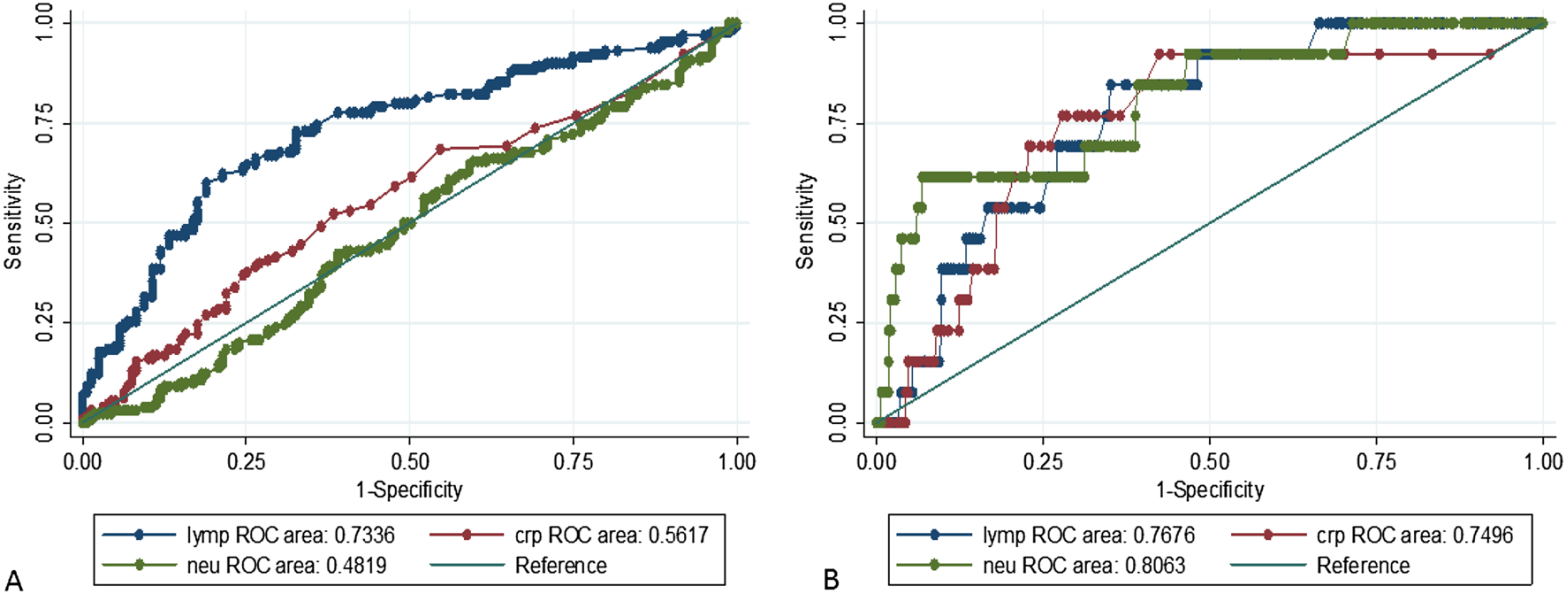
(**A**) CRP, Lymphocytes and Neutrophils response in viral agents caused URTI, (**B**) Lymphocytes and Neutrophils response in bacterial agents caused URTI.

Lymphocytes, neutrophils and CRP were used to predict the possible causative agents. Children with viral pathogens had significantly elevated lymphocytes, with normal or elevated CRP. The sensitivity of elevated lymphocytes in detecting viral pathogens was 73.4% (Fig. 3A,B).

## Discussion

### Etiologies of URTI among under five children in Mwanza city

This is the first study to establish etiologies of RTIs in the Lake Victoria zone Tanzania. Findings from this study shows that, number of viruses are responsible for RTIs among children below 5 years of age attending outpatient clinics in the city of Mwanza. The prevalence of 46.9% reported in the current study is low compared to a previous study conducted in Ifakara and Dar es Salaam, which reported prevalence of 70.5%^3^. The possible explanation for these differences could be criteria used in the enrollment of the study participants. In the previous study fever was the main inclusion criterion which was not the case in the current study. In addition, this study was conducted in different season of the year (October 2017 through February 2018) compared to the previous which was conducted from April to August and from June to December at two different sites, respectively. Viral infections have been found to be influenced by season variations^20–24^. Further studies to establish seasonality of these viruses are warranted in developing countries.

The findings in this study are comparable to the previous study in Kenya among children below 5 years of age, whereby viruses were isolated in 45% of children with RTIs^25^. In comparison to a previous study in refugee camp in Kenya the prevalence of viral infection reported in this study is low (46% vs. 66.6%)^26^. The possible explanation could be overcrowding conditions in refugee camp which has been found to facilitate transmission of RTIs viruses^27,28^.

Regarding distribution of viruses; *Rhinovirus, Adenovirus* and *Parainfluenza 3* were the commonest viral pathogens in the current study which is contrary to a previous study in Kenya^26^ whereby *Influenza A* virus, *Respiratory Syncytial virus* and *Influenza B* were the commonest. The predominance of *Rhinovirus*, *Influenza* viruses was also reported in a previous study in Tanzania^3^. The distribution of respiratory viruses is associated with climatic changes; the peak of infection usually occurs in winter period in temperate regions which is equivalent to wet season in hot climates like Tanzania^29,30^.

As documented earlier, a significant proportion of children who had viral infection presented with acute illness^27^. However, it should be noted that some studies have shown that some viral diseases may last for several weeks^27,28^ which may account for the few children in the present study who presented with chronicity.

Regarding ear bacterial infections, *Staphylococcus aureus, Pseudomonas aeruginosa*, *Serratia marcescens, Klebsiella pneumoniae, Providencia* spp. and *Streptococcus* spp. were isolated. This is contrary to the previous study whereby *Streptococcus pneumoniae* was the commonest isolate^3^. The possible explanation could be wide coverage of pneumococcal vaccines. Decrease in *Streptococcus pneumoniae* infections has been observed after introduction of the vaccine. A previous study by Mushi et al.^31^ in similar settings established that *Staphylococcus aureus* and *Pseudomonas aeruginosa* were the commonest pathogens causing CSOM among adult patients.

In the current study, only one nasopharyngeal specimens yielded bacterial isolate (*Bordetella parapertussis*) which is contrary to a previous observation by Ndossa et al.^32^ that detected *Streptococcus pneumoniae* to be colonizing the nasopharynx of children in the city of Mwanza. Moreover, the bacterial isolates in the current study were highly resistant to the readily available and the over counter antibiotics like amoxicillin, trimethoprim/sulphamethoxazole and ampicillin with majority being susceptible to ciprofloxacin ear drops. This could be explained by the fact that, ciprofloxacin is not readily used in children below 12 years of age.

### Patterns of URTI among under five children in the Mwanza city

In the current study, rhinitis was the commonest presenting disease, followed by pharyngitis and pneumonia. This is further supported by findings in the current study which reported Rhinovirus to be the commonest pathogen. A previous study^33^, documented *Rhinovirus* to cause up to 25–85% of the upper respiratory tract infections. Systemic responses of Th1 for Rhinovirus results into stimulation of specific clones of CD4 T cells and secretions of large amount of granulocytes macrophage colony stimulating factor (GM-CSF) which are also responsible for the URTI symptoms^34^. Moreover, *Rhinovirus* infection has also been associated with lower respiratory tract disease, asthma exacerbations and fatal pneumonia^33–35^. On the other hand, the spectrum of diseases in this study could be also explained by the commonly isolated viruses; *Influenza viruses*, RSV, *Parainfluenza viruses* and *Adenoviruses* which are the common viruses responsible for most of the upper respiratory diseases as previously reported^36,37^.

In the current study, children with viral pathogens had a right shift of lymphocytes with an increased sensitivity of 78.0%, with left deviation of the neutrophils or slightly raised CRP (not more than 10 mg/dl). These findings are important and could be used to predict these infections in resource limited setting and assist in decision making on the management of the patients which eventually might reduce unnecessary prescriptions of antibiotics. The observation is further supported by the fact that the majority of children were free from the initial symptoms on day eighth, underscoring that viral caused URTI have mild symptoms which tend to disappear with time^38,39^.

### Study limitations

Diagnosis of patterns of URTI was done clinically with no imaging to support the diagnosis, this might have caused misclassification. However, efforts were made to minimize this by consulting senior pediatricians whenever overlap of symptoms occurred. Another potential limitation was failure to perform multiplex PCR for all ear swabs therefore viral pathogens might have been missed in these samples.

## Conclusions and recommendations

URTI are common in children below 5 years of age and are predominantly caused by viruses. Elevated lymphocytes, normal neutrophils with elevation or normal CRP levels can predict viral causes of URTI while the raise in neutrophils and CRP are more likely to predict bacterial infections. Clinicians should suspect URTI caused by viral infections whenever the children present with runny nose with congestion, fever and dry cough. The use of antibiotics should be minimized in children with URTI symptoms since most of the symptoms disappears within a week. Further studies to determine etiologies of lower respiratory tract infections are warranted in this setting.

## Data Availability

All data are included in the manuscript. Raw data is available upon request and the request should be made to the Director of research and Innovation, Catholic University of Health and allied Sciences.
